# Supplementary figures and images for: The minimum effective concentration (MEC90) of ropivacaine for ultrasound-guided caudal block in anorectal surgery. A dose finding study
Source: PLoS One. 2021 Sep 17;16(9):e0257283. doi: 10.1371/journal.pone.0257283 (PMC8448308; doi:10.1371/journal.pone.0257283)

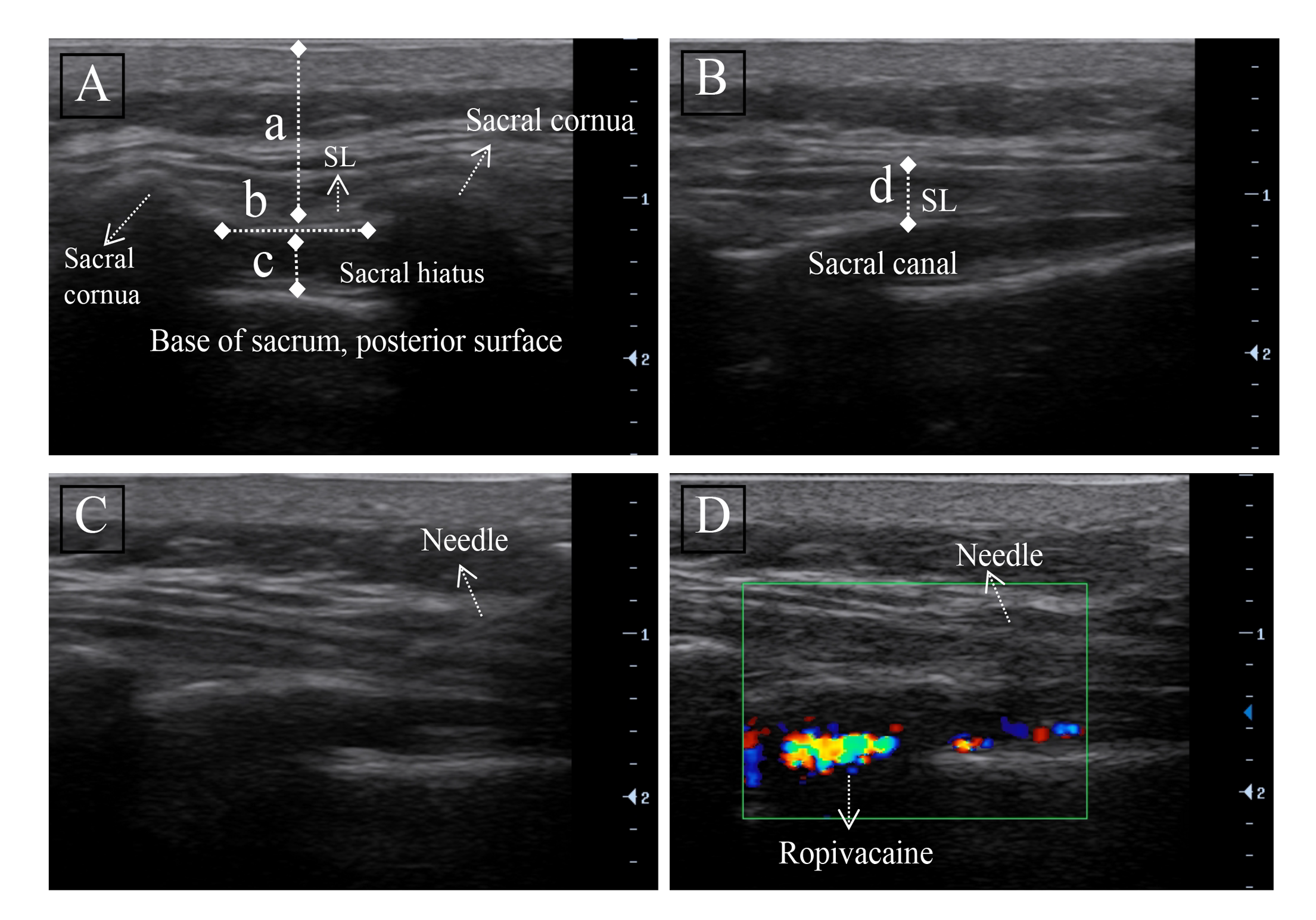

Supplement: S1 Fig — (A) Transversal ultrasound image of sacral canal. (B) Longitudinal ultrasound image of sacral canal. Measurement of the distance from anterior edge of sacrococcygeal ligament (SL) to sacrum (line c), skin to anterior edge of sacral ligament distance (line a), sacrococcygeal ligament width (b) and thickness (line d) were done. (C) Longitudinal ultrasound image of the needle in sacral canal. (D) Unidirectional flow on color doppler showing the injection of ropivacaine into sacral canal. (TIF) [file pone.0257283.s003.tif]
