# Supplementary material for: The minimum effective concentration (MEC90) of ropivacaine for ultrasound-guided caudal block in anorectal surgery. A dose finding study
Source: PLoS One. 2021 Sep 17;16(9):e0257283. doi: 10.1371/journal.pone.0257283 (PMC8448308; doi:10.1371/journal.pone.0257283)
Supplement: S1 File — (DOC) [file pone.0257283.s005.doc]

**罗哌卡因用于超声引导下骶管阻滞肛周手术患者的MEC90研究：**

**一项前瞻性药物剂量探索研究**

**研究方案**

**研究药物 罗哌卡因**

**研究负责人** 王儒蓉教授

**研究设计**  王儒蓉教授、李雪寒医师、杨明安教授、张佩医师

**研究机构 四川大学华西医院、四川大学华西医院上锦南府医院**

**版本号 V2.0**

**撰写日期 2018-12-10**

1. **研究摘要：**

**研究背景：**肛周手术常用的麻醉方法有全身麻醉和椎管内麻醉，而骶管麻醉是椎管内麻醉方式中操作简单、损伤小、对全身生理功能影响最小的一种麻醉方法，但目前尚无肛周手术骶管麻醉及术后镇痛指南。罗哌卡因具有心血管和中枢神系统不良反应轻、对运动神经阻滞较弱等特点，广泛应用于神经阻滞麻醉和术后镇痛等领域。为了保证阻滞效果，同时避免过多使用局麻药，确定局麻药的最低有效剂量(浓度×容量)具有重要的临床意义，但目前罗哌卡因用于骶管阻滞的90%最低有效容量（MEC90）尚无报道。

**研究目的：**本研究将首次使用BCD-UDM法(有偏硬币设计序贯法），研究罗哌卡因用于超声引导下骶管阻滞的MEC90，为骶管麻醉提供精准的用药指导。

**研究设计**：采用有偏硬币设计序贯法(biased coin design up-and-down sequential method，BCD-UDM)进行的药物剂量探索研究

**研究中心：四川大学华西医院上锦南府医院**。

**研究人群：**在四川大学华西上锦南府医院手术室行择期肛周手术的成年男性与女性患者

**干预措施**：选用临床常用的罗哌卡因浓度，除第一位患者使用的罗哌卡因为10ml（男性）/8ml(女性)外，接下来每一位患者接受的局麻药容量都取决于前一位患者的阻滞效果。如果前一位患者效果为“失败”(阴性反应)，则该患者所用局麻药容量增加一个单位(2ml);如果前一位患者效果为“成功”(阳性反应)，则该患者所用局麻药容量进行有偏硬币随机，有 11%(b=0.11)的概率减小一个单位(2ml)，有 89%(1-b=0.89)的概率保持不变

**主要研究终点：骶管阻滞成功与否**。

**研究预期时限**：2年

**研究注册：** Chictr.org.cn.注册号ChiCTR1900024315.

**2.背景**

围手术期疼痛的处理对肛肠手术至关重要，而骶管阻滞可通过减少使用阿片类镇痛药和其他全身性药物的使用来降低术后并发症。此外，骶管阻滞提供了术后患者自控硬膜外镇痛的可能性。罗哌卡因作为一种长效酰胺类局部麻醉药，与布比卡因相比，具有更好的感觉阻滞，但运动障碍较少，中枢神经毒性和心脏毒性也较小，这些特征使其成为肛门直肠手术，尤其是非卧床肛门手术的最佳局部麻醉药物[1]。大量研究报道罗哌卡因在骶管阻滞中应用数浓度为0.1-0.5％，体积为10-30ml，但最合适的给药方案仍然未知[2, 3]。随着骶管阻滞应用不断增加，促使学者对局麻药的体积和浓度进行研究，其中大多数研究只是简单比较两个体积或浓度组。虽然Y Li等采用Dixon-up-and-down方法探索了罗哌卡因对50％尾骶管阻滞患者的最低有效浓度（MEC50）[4]，但MEC90仍然未知，MEC90比MEC50更具有临床应用价值。我们的小组此前采用偏向硬币序贯法（BCD-UDM），确定了0.5%浓度的罗哌卡因应用于超声引导下骶管阻滞的90%最低有效容量（MEV90），男性MEV90为12.88 ml，女性为10.73ml，通过统计方法计算出男性MEV99为14ml，女性为12 ml[5].。因此，在本研究中，我们采用BCD-UDM方法，通过固定罗哌卡因容量（男性固定容量为14ml，女性固定容量为12ml）探索罗哌卡因应用于超声引导下骶管阻滞的90%最低有效浓度（MEC90）。

**3.试验目的**：

研究罗哌卡因用于超声引导下骶管阻滞的90%最低有效浓度（MEC90）,探索肛周手术患者理想的罗哌卡因剂量

**4.研究人群**

本研究采用前瞻性随机对照研究，选择在四川大学华西上锦南府医院手术室行肛周择期手术的患者，根据性别纳入女性组或男性组。

**4.1** 纳入标准:

ASA 分级I-III级

年龄 18-65 岁

BMI 18-30kg/m^2^

4.2排除标准:

超声现实骶管闭锁或狭窄（骶骨裂孔的前后径小于1.6mm）**[6];**

入选研究前3月内服用了其他试验药或者是参与了其他临床试验；

对酰胺局部麻醉药过敏，或有禁忌患者；

对试验所用麻醉、镇痛方法有禁忌症患者；

凝血功能障碍的患者或正在服用抗凝药物者

先前存在的神经病，慢性阻塞性肺病，肝或肾衰竭，脊柱疾病；

患者骶尾部局部感染或骶尾部既往外伤、手术史

口服避孕药

怀孕或哺乳期

拒绝参加研究

4.3退出标准

因各种原因不能完成实验及随访的患者

局麻药中毒的患者

**4.3脱落病例标准**

4.3.1病人自行退出（撤销同意书）；

4.3.2失访；

4.3.3研究者令其退出（出现严重不良事件）；

4.4.4失盲病例。

对于脱落病例，应在CRF表上详细记录原因并妥善保管备查，数据以最后一次评估的结局变量作为最终结果纳入统计分析。

**4.4 无效病例标准**

4.4.1研究药物未输注；

4.4.2无研究记录；

4.4.3因使用了试验中禁止使用的药物而无法进行终结局变量评价的病例。

对于无效病例，应在CRF表上详细记录原因且妥善保管备查。数据最终不纳入统计分析。

**4.5中止试验的标准**

研究进行中出现以下情况需中止试验：

4.5.1研究者发现与试验药物相关的严重安全性问题；

4.5.2方案有重大失误；

4.5.3申办方因经费或管理原因；

4.5.4行政主管部门撤销试验。

中止试验可是暂时的，也可是永久的。中止试验时，全部试验记录应予保留备查。

**5.研究总体设计**

本研究为一项采用有偏硬币序贯法的前瞻性药物剂量探索试验。单中心、前瞻性、随机、单盲研究，设有2个组（男性组和女性组）。研究由四川大学华西上锦南府医院发起，并在成都上锦南府医院麻醉科实施。

**6.样本量估算**

本项目采用有偏硬币设计序贯法(Biased coin design up-and-down sequential method，BCD-UDM法)对MEC90进行研究。为得出MEC90，根据统计学的要求，每组至少需要45(大于 40 且为 9 的倍数的最小整数)例阳性反应，总患者数无法预知，估算为 52±2 例。

**7.随机、设盲和破盲**

**7.1随机：**

我们前瞻性地纳入男性/女性患者直到出现 45 位阳性反应，并预备44个密封的信封(spss软件生成的对阳性反应的病例进行罗哌卡因容量的随机)。由与本试验执行数据管理统计分析无关的学者，在计算机上用SPSS统计软件包，随机化方法产生女性组和男性组随机编码，每组44个随机数字。

试验药物（罗哌卡因，10%）为无色透明液体，由瑞典阿斯利康有限公司提供），由指定的麻醉医生（该药剂师不参加以后的研究）根据随机数准备药物。盲底由研究中心保存直至全部试验结束。

**7.2盲法:**

7.2.1对研究人员施盲：指定一名研究协调员，负责保存和分发随机号码、准备药物以及研究人员之间的信息协调。指定一名主治医师（非盲）负责麻醉实施和记录术中信息，并进行骶管麻醉成功或失败的判定。由经过专业麻醉科医师培训的随访人员负责病人的术后随访。以上研究人员在研究期间互相不知道其余研究人员的记录内容。

7.2.2对患者施盲：所有患者术中均使用统一外观的注射器。

**7.3 破盲：**

当入组病例研究全部完成后，所有病例报告表经输入并核对无误，进行数据库锁定后进行揭盲，交统计分析人员进行统计分析。

**8.方案实施：**

**8.1 药品分发和使用：**由研究协调员根据随机数字对对阳性反应的病例进行罗哌卡因容量的随机，并在CRF表中记录该编码。

**8.2 药品使用：**试验药物均用生理盐水稀释至目标浓度。根据随机数字决定罗哌卡因使用浓度，男性病人罗哌卡因体积固定为14ml，女性则为12ml。

**8.3麻醉管理：**

患者入室后，常规监测心电图、血压、氧饱和度，鼻导管吸氧（4L/min）。建立静脉通道。嘱患者行左侧卧位，超声（M7，迈瑞，深圳，中国）评估骶管腔隙，进行骶管相关测量（骶韧带前缘距骶骨距离、皮肤距骶韧带前缘的距离、骶韧带宽度、骶韧带厚度），定位骶裂孔的位置并做标记，用20G穿刺针行骶管穿刺，当生理盐水阻力消失时，停止进针，回抽无血无脑脊液，注药针心无回弹，超声图像看到药液在骶管腔内扩散，即穿刺成功。在超声实时观察下进针，控制进针范围超过骶裂孔5mm以内，避免刺破硬脑膜。利用彩色多普勒超声的单项血流确定骶管穿刺是否成功。先给予1:20000肾上腺素的生理盐水1ml作为试验剂量，若2分钟后没有血管内注射迹象，则以0.2ml/s的速度注射罗哌卡因（10%耐乐品，阿斯利康，瑞典南泰利耶，以0.9%生理盐水稀释至0.5%浓度，无需肾上腺素）。注射完成后，取下穿刺针，消毒并更换辅料，患者回到仰卧位进行下一步评估。

罗哌卡因给药后第5分钟、10分钟、15分钟和手术结束时重复进行感觉平面的评估和运动阻滞的评估。感觉阻滞平面的评估：用26G针刺皮肤两次，评估从骶3平面到腰4平面的感觉。运动阻滞的评估采用Bromage评分，即0分为脚和膝盖完全屈曲，1分为只能移动膝盖；2分为只能移动脚踝，3分为不能移动脚或膝盖。

骶管成功的定义为：给药15分钟后肛门括约肌松弛(能通过两横指)，且患者术中无痛。若术中因疼痛或肛门括约肌松弛不够需要局麻药浸润麻醉/全身麻醉/额外给予阿片类镇痛药，则骶管阻滞失败。手术开始后静脉注射1-2mg咪达唑仑，术中给予0.5-1ug / kg·h右美托咪定以维持麻醉，使患者Ramsay评分达4-5分。术中补液6-10ml·Kg^-1^·h^-1^，若患者出现低血压/高血压，则根据血流动力学监测情况调整输液量和输液速度，维持血流动力学稳定。手术结束后评估患者感觉平面及双下肢运动评分。

**9. 资料收集**

研究者在试验开始前统一经过数据收集及结局评估有关的培训。

**9.1术前：**

9.1.1采集人口学资料，包括姓名、性别、出生日期、文化程度、受教育年限、身高、体重等；

9.1.2 术前诊断，合并疾病，麻醉ASA分级，最近一个月内的用药情况，既往手术史及麻醉史等；

**9.2 麻醉过程：**

9.2.1 记录超声测量骶管相关数据：骶韧带距骶骨Anterior edge of SL to sacrum，皮肤距骶韧带Skin to anterior edge of SL，骶韧带宽度SL width和骶韧带厚度SL thickness。

9.2.2 麻醉起效时间，骶管给药后5min、10min、15min患者感觉阻滞平面

**9.3术中**

记录手术时间、手术名称，麻醉时间、麻醉药物总用量，术中出血量、输血量、液体出入量及其他各种药物的使用情况；术中特殊情况及其处理。

**9.4术后**

9.3.1 手术结束后进行感觉阻滞平面和运动阻滞平面评估

9.3.2术后首次感觉疼痛的时间及疼痛VAS评分。采用VAS法（0=完全无痛，10=最严重疼痛）评估患者术后首次诉疼痛时的疼痛评分。

9.3.3术后24小时尿储留评分。

**10.结局指标**

**10.1 主要结局：**

骶管阻滞是否成功，根据成功与失败病例计算MEC_90_

**10.2 次要结局：**

10.2.1麻醉起效时间；

10.2.2手术时间；

10.2.3术后开始疼痛时间。

**10.3其他结局：**

10.3.1 超声测量的骶管数据：骶韧带前缘距骶骨距离、皮肤距骶韧带前缘的距离、骶韧带宽度、骶韧带厚度

10.3.2麻醉开始时感觉阻滞平面；

10.3.3手术结束时感觉阻滞平面；

10.3.4术中麻醉相关并发症发生率；

10.3.5术后运动阻滞发生率；

10.3.6术后尿潴留发生率；

10.3.7术后腰背部疼痛发生率；

10.3.8术后其他麻醉相关并发症发生率。

**11.不良事件及严重不良事件**

**11.1 不良事件**

11.1.1定义

无论与药物是否有关，凡是与研究中的任何医疗措施有关的、出现的非预期的、不利的医学事件均为不良事件。一个不良事件可以是任何非预测的和不适宜的体征（包括异常的实验室检查等）、症状或与使用产品相关的暂时性疾病。

11.1.2 本试验预期的不良事件

11.1.2.1心动过缓：心率<45次/分，或较术前基础水平下降超过30%并持续5min以上；

11.1.2.2低血压：收缩压<80mmHg，或较术前基础水平下降超过30%并持续5min以上；

11.1.2.3心动过速：心率>100次/分，或较术前基础水平增加超过30%并持续5min以上；

11.1.2.4高血压：收缩压>160mmHg，或较术前基础水平增加超过30%并持续5min以上；

11.1.2.5低氧合：脉氧饱和度<90%,或较基础水平下降超过5%。

11.1.3不良事件的处理

不良事件一旦出现，应根据临床医疗常规进行治疗，如麻醉医师或监护室医师认为有必要，可终止试验药物的输注，但需要CRF表详实记录停药时间及原因。

常见处理包括：心动过缓（静脉注射阿托品或输注异丙肾上腺素）；低血压（容量治疗及诸如麻黄碱、多巴胺、去甲肾上腺素等血管活性药的使用）；心动过速（静脉使用艾司洛尔、阿替洛尔或地尔硫卓）；高血压（静脉使用硝普钠、乌拉地尔、地尔硫卓等血管活性药）；低氧合（增加吸入氧浓度或调整呼吸机参数以及胸部物理治疗）。

11.1.4记录

任何发生的不良事件均应记录，包括种类、诊断时间、处理、持续时间、结局等。

不良事件随访至消失或治疗终结。

**11.2 严重不良事件**

11.2.1 定义

严重不良事件是指非预期的导致病人死亡、危及病人生命、永久性伤残或延长住院时间的其他严重情况的医学事件。

骶管麻醉相关严重不良事件主要有：局麻药中毒（中枢兴奋和惊厥，低血压、循环衰竭、甚至心跳停止）、局麻药过敏（皮肤粘膜水肿、荨麻疹、哮喘、低血压或休克等症状）、全脊麻（全部脊神经支配的区域均无痛觉、低血压、意识丧失及呼吸停止）。

11.2.2严重不良事件的处理

如出现严重不良事件，除应积极治疗或抢救外，应在获知后24小时内以电话或传真形式通知临床研究负责人和伦理委员会。如发生与治疗相关的死亡事件，应立即停止临床试验，尽快上报伦理委员会，并详细记录和妥善保存有关资料。

所有严重不良事件都应当追踪，并及时记录不良事件的出现、类别、发生时间、程度、持续时间及转归。直到得到妥善解决或病情稳定。

**11.3 不良事件的监测期限**

从实验药物开始输注至术后24h。但不良事件一旦发生，应监测至其消失或治疗终结。

**12.数据管理**

12.1研究者根据受试者的原始观察记录，将数据及时、完整、正确的载入病例报告表；

12.2监查员监查试验的进行是否遵循试验方案。病例报告表经监查员签字后，及时送交临床试验数据保管员；

12.3病例报告表在按要求完成数据录入和核查后（一人录入，另一人核查），按编号的顺序归档保存。

12.4 数据监察由四川大学华西医院上锦南府医院临床研究伦理委员会进行。

**13.统计学分析**

**13.1 一般原则：**

计量资料根据数据分布的形态采用均值（标准差）或中位数（四分位间距）表示；计数资料用例数（百分比）表示。所有的统计检验均采用双侧检验，P值小于或等于0.05将被认为所检验的差别有统计意义。

**13.2入组及完成情况：**

总结入组及完成病例。

**13.3 一般信息与基线特征：**

对患者的人口学信息、用药史、合并其他疾病史等进行统计描述。对于男性组与女性组的基线数据比较，计量资料先进行Kolmogorove-Smirnov正态性检验，使用Levene检验来评估方差齐性。若符合正态分布且方差齐，使用独立样本t检验，对于不符合正态分布或方差不齐的计量资料采用Kruskale-Wallis检验比较；计数资料采用卡方检验／Fisher精确检验。P <0.05被定义为具有统计学意义。

**13.4. 疗效评价**

13.4.1主要结局评价：

对两组骶管成功病例进行统计描述。运用R 统计软件，通过保序回归(isotonic regression)计算出 MEC90，通过 2000 重复样本的自助算法(bootstrapping)计算出结果的95%可信区间。

13.4.2 次要结局评价

13.4.2.1 麻醉开始时感觉阻滞平面：统计每个平面病例数；

13.4.2.2 手术结束时感觉阻滞平面：统计每个平面病例数；

13.4.2.3 术中麻醉相关并发症（低血压、心动过缓等）发生率：组间采用卡方检验；

13.4.3 其他指标评价

13.4.3.1 术后运动阻滞发生率：采用卡方检验；

13.4.3.2术后尿潴留发生率：采用卡方检验；

13.4.3.3术后腰背部疼痛发生率：采用卡方检验；

13.4.3.4术后其他麻醉相关并发症发生率：采用卡方检验；

13.4.3.5骶韧带前缘距骶骨距离：采用t检验或Kruskale-Wallis检验；

13.4.3.6皮肤距骶韧带前缘的距离：采用t检验或Kruskale-Wallis检验；

13.4.3.7骶韧带宽度：采用t检验或Kruskale-Wallis检验；

13.4.3.8骶韧带厚度：采用t检验或Kruskale-Wallis检验。

**14．质量控制**

14.1研究开始前向研究人员和医务人员详细解释研究方案。研究期间必须必须严格遵循研究方案；

14.2研究人员应完整、详细、准确的填写病例报告表。临床试验中所有观察到的结果和异常发现，均应及时加以认真核实、记录，保证数据的可靠性；

14.3试验中各种检查项目所使用的各种仪器设备，均应有严格的质量标准，并确保正常工作；

14.4统计分析由专业生物统计学的人员完成；

14.5所有结论应基于原始数据得出。

**15伦理学要求**

15.1遵循赫尔辛基宣言和中国有关临床试验管理规范进行临床试验。在试验开始之前，由临床研究伦理委员会批准试验方案后方可实施临床试验；

15.2术前研究人员到病房向符合筛选条件的患者或/及其委托人充分说明本研究的研究目的、方法及其可能带来的益处和危害，并详细解答患者及家属的疑问，给予其充分的考虑时间，在完全自愿的前提下请患者或/及其委托人签署知情同意书。对术前已明确存在痴呆等神经精神疾病或无自主行为能力的患者，应具备委托人签字。知情同意书作为研究文件保存；

15.3所有与研究相关的个人信息均属保密资料，不允许拷贝。除参与本临床研究的研究人员、伦理委员会、研究单位相关管理部门外，不得向其他任何人、任何单位透露。

**16资料保存**

研究者、申办者应当按照GCP要求对临床试验的文件和资料进行妥善保存。

**17预期进度**

17.1临床试验期：6-12个月。主要包括入选患者的筛选、入选、治疗及随访；

17.2数据处理和资料总结期：6-12个月。主要对临床试验所得资料进行统计学处理、资料的总结。

**18．参考文献**

1. Simpson D, Curran MP, Oldfield V, Keating GM. Ropivacaine: a review of its use in regional anaesthesia and acute pain management. Drugs. 2005;65(18):2675-717. Epub 2006/01/06. doi: 10.2165/00003495-200565180-00013. PubMed PMID: 16392884.

2. Gan S, Song L, Chen W, Feng Z, Li Y, Zhang J, et al. Strength and sensation after epidural ropivacaine in men and women. Anaesthesia. 2015;70(9):1060-5. Epub 2015/04/29. doi: 10.1111/anae.13085. PubMed PMID: 25919788.

3. Wiegele M, Marhofer P, Lönnqvist PA. Caudal epidural blocks in paediatric patients: a review and practical considerations. British journal of anaesthesia. 2019;122(4):509-17. Epub 2019/03/13. doi: 10.1016/j.bja.2018.11.030. PubMed PMID: 30857607; PubMed Central PMCID: PMCPMC6435837.

4. Li Y, Zhou Y, Chen H, Feng Z. The effect of sex on the minimum local analgesic concentration of ropivacaine for caudal anesthesia in anorectal surgery. Anesthesia and analgesia. 2010;110(5):1490-3. Epub 2010/03/23. doi: 10.1213/ANE.0b013e3181d6bade. PubMed PMID: 20304981.

5. Xuehan Li PZ, Rurong Wang. The minimum effective volume (MEV90) of ropivacaine for ultrasound-guided caudal block in anorectal surgery. European journal of anaesthesiology. 2020;37(e-Supplement 58):7-. Epub June 2020.

6. Chen CP, Wong AM, Hsu CC, Tsai WC, Chang CN, Lin SC, et al. Ultrasound as a screening tool for proceeding with caudal epidural injections. Arch Phys Med Rehabil. 2010;91(3):358-63. Epub 2010/03/20. doi: 10.1016/j.apmr.2009.11.019. PubMed PMID: 20298824.

7. Stylianou M, Proschan M, Flournoy N. Estimating the probability of toxicity at the target dose following an up-and-down design. Statistics in medicine. 2003;22(4):535-43. Epub 2003/02/19. doi: 10.1002/sim.1351. PubMed PMID: 12590412.

8. Stylianou M, Flournoy N. Dose finding using the biased coin up-and-down design and isotonic regression. Biometrics. 2002;58(1):171-7. Epub 2002/03/14. doi: 10.1111/j.0006-341x.2002.00171.x. PubMed PMID: 11890313.

**The minimum effective concentration (MEC90) of ropivacaine for ultrasound-guided caudal block in anorectal surgery:**

**a prospective does-finding study using biased coin design up-and-down sequential method**

**Study protocol**

**Study Drug**: Ropivacaine for injection

**Principal Investigator**: Rurong Wang, MD, PhD

**Study Design**: Rurong Wang, MD, PhD; Xuehan Li, MD; Mingan Yang, PhD and Pei Zhang, MM

**Name of Institute**: Department of Anesthesiology (Rurong Wang, MD, PhD, Xuehan Li, MD and Pei Zhang, MM), West China Hospital of Sichuan University, Chengdu 610041, China; Division of Biostatistics & Epidemiology (Mingan Yang, PhD), School of Public Health, San Diego State University, San Diego, CA, 92182, USA

**Version: 2.0**

**Date of version: December 10, 2018**

**1. Abstract**

**1.1. Background**

 Caudal epidural block (CEB) provides reliable anesthesia for adults undergoing anorectal surgery. Despite the widely utilization, the minimum effective concentration (MEC) of ropivacaine for CEB remains unknown.

**1.2. Objectives**

To estimate MEC of ropivacaine 0.5% for CEB in anorectal surgery.

**1.3. Study design**

A prospective dose-finding study using biased coin design up-and-down sequential method

**1.4. Setting**

Chengdu ShangjinNanfu Hospital, from October 2019 to January 2020

**1.5. Patients**

45 males and 45 females scheduled for anorectal surgery who are subjected to successful caudal block.

**1.6. Intervention**

Two independent biased coin design up-and down trials by genders will be conducted. The concentration of ropivacaine administered to the first patient of male and female are 0.25% with fixed volume of 14ml for male and 12ml for female patients based on our previous study. In case of failure, the concentration will be increased by 0.05% in the next subject. Otherwise, the next subject will be randomized to a concentration 0.05% less with a probability of 0.11, or the same concentration with a probability of 0.89. Success is defined as complete sensory blockade of perineal area 15 min after the block evidenced by the presence of a lax anal sphincter and pain-free surgery.

**1.7. Main outcome**

The MEC of ropivacaine to achieve a successful CEB in 90%(MEC90) of the patients.

**1.8. Predicted duration of the study**

2 years.

**1.9. Trial registration**

The study was registered with Chictr.org.cn. Identifier: No. ChiCTR 1900024315

**2. Background**

Perioperative pain management is vital for anorectal surgery, and caudal block may lower the postoperative complication by reducing the use of opioid analgesics and other systemic drugs. Besides, cauda block provides the possibility of patient-controlled epidural analgesia postoperatively with few motor blocks.

Ropivacaine, as a long-acting amide local anesthetic, shows a better sensory block while presents less motor block than bupivacaine and less central nerve toxicity and cardiotoxicity, and these characteristics make it the optimal reginal anesthetic agent for anorectal surgery, especially for ambulatory anorectal surgery[1]. Plenty of studies had reported how ropivacaine applied in caudal block, mostly with concentration of 0.1-0.5% and volume of 10-30ml, but the most appropriate dosage regiment remains unknown. The rising application of caudal block urges studies in local anesthetic volume and concentration, most of which were just simple comparisons of two volume or concentration groups. Although Y Li et al. reported minimum effective concentration in 50% of patients (MEC50) of ropivacaine in caudal block by using Dixon’s up-and-down method, MEC90 remains unknown which might be of clinical sense. Our group has identified minimum effective volume in 90% of patients(MEV90) of ropivacaine with a fixed concentration 0.5% for caudal block in adults by applying a biased coin design (BCD) up-and-down method (UDM) (BCD-UDM) and reported a volume of 12.88 ml and 10.73ml ropivacaine 0.5% provided a successful caudal block in 90% of the male and female patients respectively and a volume of 14ml for male and 12 ml for female would be successful in 99% of the patients[5]. Hence, we are exploring MEC90 of ropivacaine in caudal block for anorectal surgery with fixed volume of 14ml for male and 12ml for female patients in the present study.

**3. Purpose of the study**

To estimate MEC of ropivacaine 0.5% to achieve a successful CEB in 90%(MEC90) of the patients.

**4. Recruitment of participants**

Patients undergoing hemorrhoidectomy or anal fistula resection surgery or anorectal polypectomy in Chengdu Shangjin Nanfu Hospital were prospectively enrolled. Potential participants will be screened the day before surgery or, for those who will undergo surgery on Monday, on Friday before surgery.

**4.1. Inclusion criteria**

4.1.1. Aged between 18 and 65 years old

4.1.2. American Society of Anesthesiologists (ASA) status I to III

4.1.3 Body mass index between 18 and 30 kg/m^2^

4.1.4 Scheduled to undergo hemorrhoidectomy or anal fistula resection surgery or anorectal polypectomy

**4.2. Exclusion criteria**

Patients who meet any of the following criteria will be excluded:

4.2.1. inability to consent to the study

4.2.2. Ultrasound showed that the sacral canal was narrow or occlusive (the antero-posterior diameter of the sacral hiatus less than 1.6mm[6];

4.2.3. other test drugs were taken within 3 months before the study was selected or participated in other clinical trials;

4.2.4. allergic to amide local anesthetics, or contraindicated; patients with coagulopathy or taking anticoagulant;

4.2.5. preexisting neuropathy, chronic obstructive pulmonary disease, hepatic or renal failure, spinal disease;

4.2.6. local infection in the patient’s caudal region;

4.2.7. prior surgery or injury in the sacrococcygeal region;

4.2.8. oral administration of contraceptives during the previous week;

4.2.9. pregnancy or lactation;

4.2.10. Any other conditions that are considered unsuitable for study participation.

**4.3. Criteria of drop out**

4.3.1. Withdrawn consents by the participants themselves;

4.3.2. Loss to follow-up;

4.3.3. Ordered to exit by the investigators or attending physicians (occurrence of severe adverse events or severe complications);

4.3.4. Cases of unmasked blindness.

For drop out cases, the detailed reasons will be recorded and the primary therapeutic effects recorded in the last time will be regarded as the final results. The Case Report Forms (CRFs) of these cases will be preserved for future reference.

**4.4. Criteria of rejection**

Enrolled cases who meet any of the following criteria will be excluded from further per protocol analysis.

4.4.1. Study drug is not administered;

4.4.2. No research record;

4.4.3. Unable to evaluate the effectiveness and safety because of the use of any prohibited drugs.

For rejected cases, the detailed reasons will be recorded and CRFs will be preserved for reference. The results of these cases will be excluded for further analysis of therapeutic effects.

**4.5. Criteria of study interruption**

Study will be interrupted in the following situations:

4.5.1 The investigator found serious safety problems related to the experimental drug;

4.5.2. Serious mistake found in the protocol;

4.5.3. Fund or management problem of the investigators;

4.5.4. Study cancelled by the administrative authority.

Study interruption may transient or permanent. All recorded CRFs will be preserved for reference in case of study interruption.

**5. Study Design**

5.1. This is a prospective dose-finding study using biased coin design up-and-down sequential method (BCD-UDM);

5.2. It is coordinated by the Department of Anesthesiology of West China Hospital, Sichuan University;

5.3. The study will be conducted in Chengdu Shangjin Nanfu Hospital.

**6. Sample Size Estimation**

To estimate MEC90, a minimum of 45 positive responses were required[7, 8]. Thus, we will prospectively recruit patients until 45 successful blocks were accomplished, and a set of 44 sealed envelopes for each gender (with the random concentration assignments inside for successful blocks). The envelopes will be prepared by a resident who will take no further part in the study.

**7. Randomization, blinding and unmask of blinding**

**7.1. Randomization**

7.1.1. A biostatistician who does not participate in data management and statistical analysis will generate 44 random numbers for each gender using the SPSS statistical package.

7.1.2. The test drug (ropivacaine, 10%) is a colorless and transparent liquid, provided by AstraZeneca Co., Ltd. of Sweden), and the drug will be prepared by an anesthesiologist who does not participate in the rest of the study will encode the study drugs according to the randomization sequence according to random numbers. The randomization results will be sealed in sequentially numbered letters and stored at the site of investigation until the end of the study.

**7.2. Blinding**

7.2.1 Blind the researchers: appoint a research coordinator who is responsible for keeping and distributing random numbers, preparing drugs, and coordinating information among researchers. Appoint an experienced anesthesiologist (unblinded to the success of CEA) to be responsible for the implementation of anesthesia, record the information during the operation, and determine the success or failure of caudal block. Follow-up personnel trained by professional anesthesiologists are responsible for the postoperative follow-up of patients. The above researchers did not know each other's records during the research period.

7.2.2 Blind the patient: All syringes and injection pumps used for study drug administration were same in external appearance.

**7.3.** **Unmask of blinding**

After each trial patient’s study is completed and all data are inputted into the database and checked without mistakes, the database will be locked up and the blindness will be unmasked. The database will then be sent to a biostatistician for statistical analysis.

**8. Intervention and anesthesia management**

**8.1. Distribution of study drugs**

The research coordinator will randomize the ropivacaine concentration of positive cases based on random numbers, and record the code in the CRF table.

**8.2 Drug use**

Ropivacaine is diluted to targeted concentration with saline. The volume of ropivacaine will be fixed at 14ml for male and 12ml for female patients.

**8.3. Anesthesia management**

8.3.1. No opioid analgesics or other analgesics was administered before or during the operation.

8.3.2. Intraoperative monitoring: includes electrocardiogram, pulse [oxygen saturation](app:ds:oxygen saturation), non-invasive blood pressure, and volume of infusion. Other monitoring (invasive arterial pressure, central venous pressure, etc.) will be performed when necessary. Fluid administration was controlled to 6-10 ml· kg-1·h-1 in operation room.

8.3.3. Anesthesia

After preparation, patients will be placed in a left lateral position, and ultrasound (M7, Mindray, Shenzhen, China) guided caudal block will be performed by experienced anesthetists using the same ultrasound as follows(5,6): At first, the probe is placed in the middle of the sacrum and the transverse view showing the superficial sacrococcygeal ligament in between two sacral cornua, and the deeper sacral bone base. Between the sacrococcygeal ligament (SL) and the sacral bone is the sarcral hiatus, where the needle will be inserted to. Measurement of the distance from anterior edge of sacrococcygeal ligament to sacrum (line c), skin to anterior edge of sacral ligament distance will be done. Then, the probe is turned 90 degrees to get longitudinal view and thickness of sacrococcygeal ligament (line d) will be measured and then a 20G intravenous catheter with an inner stylet is inserted through the sacrococcygeal ligament into the sacral hiatus. The caudal space is identified with the loss of resistance technique using saline. The block needle will be visualized in real time to keep the advancement of needle tip beyond the apex of sacral hiatus limited to 5 mm to avoid dural puncture. Unidirectional flow on color doppler was utilized to identify the success of a caudal block. After negative aspiration,1 ml of a solution containing 5ug epinephrine is administrated as a test dose. If after 2 min there is no evidence of intravascular injection, ropivacaine (10% Naropin; AstraZeneca, Sodertalje, Sweden) diluted with 0.9% w/v saline to achieve 0.5% concentration without epinephrine will be injected at the rate of 0.2 ml/s. After injection, the needle is removed and the patient will be turned to supine for further assessment.

8.3.4. Evaluation of anesthesia onset

Block onset is evaluated by pinprick around the perineal area (S3 dermatome) and the existence of flaccid anal sphincter. We defined the effective caudal block only if the presence of a lax anal sphincter 15 minutes after the caudal injection and the patient had pain-free surgery without the need for rescue blocks including supplemental opioids, general anesthesia or local infiltration by the surgeon. After completion of the assessment at 15 min indicating the success of CEB, 1-2mg midazolam and 0.5-1ug/kg·h dexmedetomidine were administered intravenously for maintenance of anesthesia. The block was considered ineffective if there was pain during surgery or the presence of a tight anal sphincter, and the patient received rescue blocks.

8.3.5. Evaluation of sensory block level

Sensory block level was evaluated as follows: sensitivity to pinprick was tested from sacral segment 3 (S3) to even lumbar segment 4 (L4) dermatomes by pricking the skin twice with a 26 G needle. The pinprick test was repeated at 5, 10, 15 min following administration of ropivacaine and at the end of surgery.

8.3.6. Evaluation of motor block

Motor block was evaluated according to the Bromage scale (0=full flexion of feet and knees, 1=just able to move knees, 2=able to move feet only, and 3=unable to move feet or knees) performed at 5, 10, 15 min following administration of ropivacaine and at the end of surgery.

8.3.7. Rescue block

We defined the effective caudal block only if the presence of a lax anal sphincter 15 minutes after the caudal injection and the patient had pain-free surgery without the need for rescue blocks including supplemental opioids, general anesthesia or local infiltration by the surgeon.

**9. Data collection**

Investigators performing data collection and assessment will be trained and qualified before the study.

**9.1. Preoperative data**

9.1.1. Demographic data, including gender, height, weight, date of birth, education, etc., will be collected;

9.1.2. Preoperative diagnosis, comorbidity, history of medication (especially within one month), history of previous anesthesia and surgery will be collected.

**9.2 Anesthesia data**

9.2.1 Record related data of ultrasonic measurement of sacral canal: anterior edge of SL to sacrum, skin to anterior edge of SL, SL width and SL thickness.

9.2.2 Onset time of anesthesia, sensory block level at 5min, 10min, 15min after sacral canal administration and the end of surgery.

**9.3. Intraoperative data**

9.3.1. Duration of anesthesia, type and dose of drugs used during anesthesia;

9.3.2. Fluid balance and transfusion of blood products;

9.3.3. Type and duration of surgery;

9.3.4. Special conditions and treatment during the operation.

**9.4. Postoperative data**

9.4.1. Evaluation of sensory block level after the operation;

9.4.2. Evaluation of motor block level after the operation.

9.4.3. Time to feel pain for the first time after operation and pain VAS score. The VAS method (0=completely painless, 10=most severe pain) was used to evaluate the pain score when the patient first complained of pain after surgery.

9.4.5. The 24-hour urine retention score after surgery;

9.4.6. Occurrence of complications (pulmonary complications, acute kidney injury [, cardiac events, cerebrovascular events, infections, etc.) during the first 5 days after surgery.

**10. Outcomes**

**10.1 Main outcome:**

Whether the sacral canal block is successful, MEC90 is calculated based on the success and failure cases.

**10.2. Secondary outcomes**

10.2.1 Onset time of anesthesia;

10.2.2 Operation time;

10.2.3 Time to start pain after operation.

**10.3. Additional outcomes**

10.3.1 Sacral canal data measured by ultrasound: distance from the anterior edge of the sacral ligament to the sacrum, distance from the skin to the anterior edge of the sacral ligament, width of the sacral ligament, thickness of the sacral ligament

10.3.2 Sensory block level at the beginning of anesthesia;

10.3.3 Sensory block level at the end of the operation;

10.3.4 The incidence of intraoperative anesthesia-related complications;

10.3.5 Incidence of postoperative motor block;

10.3.6 Incidence of postoperative urinary retention;

10.3.7 The incidence of postoperative back pain;

10.3.8 The incidence of other anesthesia-related complications after surgery;

10.3.7 The incidence of postoperative back pain;

10.3.8 The incidence of other anesthesia-related complications after surgery.

**11. Adverse events and severe adverse events**

**11.1.** **Adverse events**

**11.1.1. Definition**

An adverse event indicates any unpredictable, unfavorable medical event that is associated with any medical intervention and occurs during the study period. It can be related to the study drug administration or otherwise. It can manifest as any uncomfortable signs (including abnormal laboratory findings), symptoms or transient morbidity;

**11.1.2. Predicted adverse events in this study**

11.1.2.1. Bradycardia: heart rate < 45 beat per minute or a decrease of more than 30% from baseline (average value in the ward), and lasting for at least 5 minutes;

11.1.2.2. Hypotension: systolic blood pressure < 80 mmHg or a decrease of more than 30% from baseline, and lasting for at least 5 minutes;

11.1.2.3. Tachycardia: heart rate > 100 beats per minute or an increase of more than 30% from baseline, and lasting for at least 5 minutes;

11.1.2.4. Hypertension: systolic blood pressure > 160 mmHg or an increase of more than 30% from baseline, and lasting for at least 5 minutes;

11.1.2.5 Desaturation: pulse oxygen saturation < 90% or a decreased of more than 5% (absolute value) from baseline.

**11.1.3. Management**

11.1.3.1. Therapy will be provided according to routine clinical practice;

11.1.3.2. The study drug infusion rate can be decreased temporarily or permanently, or the study drug infusion can be stopped temporarily or permanently if considered necessarily by the attending anesthesiologists. The time and reasons of study drug interruption will be recorded in the CRFs;

11.1.3.3. Bradycardia: administration of medication (atropine iv bolus and/or isoprenaline iv infusion only);

11.1.3.4. Hypotension: intravenous fluid bolus, administration of medication (ephedrine/phenylephrine iv bolus, dopamine/dobutamine/epinephrine/norepinephrine, iv infusion);

11.1.3.5. Tachycardia: administration of medication (esmolol/diltiazem/atenolol iv infusion);

11.1.3.6. Hypertension: administration of medication (sodium nitroprusside/ditiazem/ urapidil/nicardipine iv bolus/infusion);

11.1.3.7. Desaturation: administration of oxygen (for patients without endotracheal intubation), adjustment of ventilator setting (for patients with endotracheal intubation), and/or physical therapy.

**11.1.4. Record**

11.1.4.1. Any adverse event should be documented, including occurrence, type, time of diagnosis, management, duration of persistence, and sequelae;

11.1.4.2. Any adverse event should be followed up until it is completely resolved or therapy termination.

**11.2. Severe adverse events**

**11.2.1. Definition**

A severe adverse event indicates any unpredictable medical events that lead to death, threat of life, prolonged length of hospital stay, persistent disability or dysfunction, or other severe event.

Severe adverse events related to sacral anesthesia mainly include: local anesthetic poisoning (central excitement and convulsions, hypotension, circulatory failure, and even cardiac arrest), local anesthetic allergy (skin mucous membrane edema, urticaria, asthma, hypotension or shock and other symptoms) , General spinal anesthesia (all areas innervated by spinal nerves are without pain, hypotension, loss of consciousness and respiratory arrest).

**11.2.2. Management**

In case of any severe adverse events, the study drug infusion will be stopped and treatment will be initiated immediately.

**11.2.3. Record and report**

11.2.3.1. In case of any severe adverse event, apart from active treatment and record as above, the principal investigator and the Ethics Committee will be informed within 24 hours in written report;

11.2.3.2. In case of study drug related death, immediately stop the clinical trial, report the event to the Ethics Committee as soon as possible, record in detail and carefully preserve the related documents;

11.2.3.3. Any severe adverse event must be followed up until it is completely resolved or when therapy is ended.

**11.3. Monitoring and follow up**

11.3.1. Adverse events and severe adverse events will be monitored from start of study drug infusion until 24 hours after surgery;

11.3.2. If an adverse event or a severe adverse event occurs, it will be followed up until complete resolution or therapy termination.

**12. Data management**

12.1. Investigators should promptly, completely, and correctly record data in the CRF according to original observation;

12.2. Supervisors will monitor if the study is carried out according to the protocol. The completed CRFs, after signed by the supervisor, will be sent to an investigator who is responsible for data management;

12.3. Data input will be performed by one investigator and checked out by another independent researcher. CRFs will be stored in sequence order;

12.4. Data management will be inspected by the Clinical Research Ethics Committee of Chengdu Shangjin Nanfu Hospital.

**13. Statistical analysis**

**13.1. General principles**

13.1.1. Numeric variables will be presented as mean (standard deviation) or median (minimum, maximum; or interquartile range). Categorical variables will be presented as number of cases (percentage);

13.1.2. Two-tailed tests will be used in all statistical analysis, and p values of less than 0.05 will be considered to be of statistical significance (unless otherwise indicated).

**13.2. Patient recruitment and drop-out situation**

The status of patient recruitment and drop-out will be summarized and listed.

**13.3. Demographics and baseline characteristics**

13.3.1. Demographic information and baseline characteristics will be presented;

13.3.2. Comparison of baseline numeric variables (such as age, BMI etc.) between groups will be performed with independent sample t-test, and the Kruskal-Wallis test is used for the measurement data that does not conform to the normal distribution or the variance is uneven; . Comparison of categorical variables (such as presence of a comorbidity etc.) between groups will be performed with chi-square test or Fisher exact test.

**13.4. Effectiveness evaluation**

**13.4.1. Evaluation of primary outcome**

The two groups of successful caudal block cases will be presented. Using R statistical software, MEC90 is calculated by isotonic regression, and the 95% confidence interval of the result is calculated by bootstrapping of 2000 repeated samples.

**13.4.2. Evaluation of secondary outcomes**

13.4.2.1 Sensory block level at the beginning of anesthesia: count the number of cases of each level;

13.4.2.2 Sensory block level at the end of the operation: count the number of cases of each level;

13.4.2.3 The incidence of intraoperative anesthesia-related complications (hypotension, bradycardia, etc.): Chi-square test was used between groups;

**13.4.3 Evaluation of other outcomes**

13.4.3.1 The incidence of postoperative motor block: using chi-square test;

13.4.3.2 Incidence of postoperative urinary retention: using chi-square test used;

13.4.3.3 Incidence of low back pain after operation: using chi-square test;

13.4.3.4 The incidence of other postoperative complications related to anesthesia: using chi-square test;

13.4.3.5 Distance from the anterior edge of the sacral ligament to the sacrum: use t-test or Kruskal-Wallis test;

13.4.3.6 The distance between the skin and the anterior edge of the sacral ligament: use t test or Kruskal-Wallis test;

13.4.3.7 Width of sacral ligament: use t test or Kruskal-Wallis test;

13.4.3.8 Sacral ligament thickness: use t test or Kruskal-Wallis test.

**14. Quality control**

14.1. Trial protocol will be thoroughly explained to all investigators/care givers before the start of the trial. The trial protocol must be strictly adhered throughout the trial period;

14.2. All expected and unexpected findings will be documented promptly and correctly in order to guarantee the reliability of the values;

14.3. The monitors and instruments that are used during the study period will be checked and corrected regularly in order to guarantee their normal work;

14.4. Data analysis will be performed by the biostatisticians and investigators;

14.5. Any conclusions must be derived from the original data.

**15. Ethical issues**

15.1. Helsinki declaration and Chinese guidelines of Good Clinical Practice will be strictly followed. The study protocol must be approved by the Ethics Committee before the study can be started;

15.2. For every potential participant, investigators have the responsibilities to fully explain the study purpose, procedures, as well as possible risks in a written informed manner. They must let every potential participant know that he/she has the right to withdraw his/her consent at any time during the study period. Every potential participant must be given a written informed consent. Every participant or the authorized surrogate of the patient must sign the consent before they can be enrolled in the study. Written informed consents will be kept as a part of the clinical trial documents;

15.3. Personal information of all participants will be kept secret. Results of the study will be published in the form of theses, but personal information (including name, age, etc.) will be kept secret.

**16. Preservation of documents**

Investigators will carefully preserve all documents and data of the clinical trial according to the Good Clinical Practice requirement.

**17. Anticipated schedule**

17.1. Recruitment of participants: 6-12 months;

17.2. Data analysis and draft manuscript: 6-12 months.

**18.References**

1. Simpson D, Curran MP, Oldfield V, Keating GM. Ropivacaine: a review of its use in regional anaesthesia and acute pain management. Drugs. 2005;65(18):2675-717. Epub 2006/01/06. doi: 10.2165/00003495-200565180-00013. PubMed PMID: 16392884.

2. Gan S, Song L, Chen W, Feng Z, Li Y, Zhang J, et al. Strength and sensation after epidural ropivacaine in men and women. Anaesthesia. 2015;70(9):1060-5. Epub 2015/04/29. doi: 10.1111/anae.13085. PubMed PMID: 25919788.

3. Wiegele M, Marhofer P, Lönnqvist PA. Caudal epidural blocks in paediatric patients: a review and practical considerations. British journal of anaesthesia. 2019;122(4):509-17. Epub 2019/03/13. doi: 10.1016/j.bja.2018.11.030. PubMed PMID: 30857607; PubMed Central PMCID: PMCPMC6435837.

4. Li Y, Zhou Y, Chen H, Feng Z. The effect of sex on the minimum local analgesic concentration of ropivacaine for caudal anesthesia in anorectal surgery. Anesthesia and analgesia. 2010;110(5):1490-3. Epub 2010/03/23. doi: 10.1213/ANE.0b013e3181d6bade. PubMed PMID: 20304981.

5. Xuehan Li PZ, Rurong Wang. The minimum effective volume (MEV90) of ropivacaine for ultrasound-guided caudal block in anorectal surgery. European journal of anaesthesiology. 2020;37(e-Supplement 58):7-. Epub June 2020.

6. Chen CP, Wong AM, Hsu CC, Tsai WC, Chang CN, Lin SC, et al. Ultrasound as a screening tool for proceeding with caudal epidural injections. Arch Phys Med Rehabil. 2010;91(3):358-63. Epub 2010/03/20. doi: 10.1016/j.apmr.2009.11.019. PubMed PMID: 20298824.

7. Stylianou M, Proschan M, Flournoy N. Estimating the probability of toxicity at the target dose following an up-and-down design. Statistics in medicine. 2003;22(4):535-43. Epub 2003/02/19. doi: 10.1002/sim.1351. PubMed PMID: 12590412.

8. Stylianou M, Flournoy N. Dose finding using the biased coin up-and-down design and isotonic regression. Biometrics. 2002;58(1):171-7. Epub 2002/03/14. doi: 10.1111/j.0006-341x.2002.00171.x. PubMed PMID: 11890313.
