# Supplementary material for: The minimum effective concentration (MEC90) of ropivacaine for ultrasound-guided caudal block in anorectal surgery. A dose finding study
Source: PLoS One. 2021 Sep 17;16(9):e0257283. doi: 10.1371/journal.pone.0257283 (PMC8448308; doi:10.1371/journal.pone.0257283)
Supplement: Attachment — Submitted filename: renamed_ce123.docx [file pone.0257283.s006.docx]

**COMMENTS – Manuscript PONE-D-20-29940**

Title: “The minimum effective concentration (MEC90) of ropivacaine for ultrasound-guided caudal block in anorectal surgery. A does finding study”

**Important note: This review pertains only to ‘statistical aspects’ of the study and so ‘clinical aspects’ [like medical importance, relevance of the study, ‘clinical significance and implication(s)’ of the whole study, etc.] are to be evaluated [should be assessed] separately/independently. Further please note that any ‘statistical review’ is generally done under the assumption that (such) study specific methodological [as well as execution] issues are perfectly taken care of by the investigator(s). This review is not an exception to that and so does not cover clinical aspects {however, seldom comments are made only if those issues are intimately / scientifically related & intermingle with ‘statistical aspects’ of the study}. Agreed that ‘statistical methods’ are used as just tools here, however, they are vital part of methodology [and so should be given due importance].**

**COMMENTS:** Because this is a dose-finding study, there are not much to comment on [in context of statistical review]. For this purpose, design used (biased coin design up-and-down sequential method) is perfectly alright {though I do not have any first-hand experience of using this design, on the basis of as much I know, I can dare to say this}. Randomization procedure used [lines 14-17], I guess, is known as ‘two-arm-bandit problem in play the winner allocation rule’. Please confirm if my information is correct, kindly inform otherwise.

You might have rightly used references 7 & 8 [published in very good journals, namely Statistics in medicine and Biometrics], to estimate assignment ‘probabilities’, the explanation given in lines 165-7 [If a patient had a successful block, the next subject was randomized with probability b=0.11 to the next lower concentration and 1-b=0.89 to the same concentration] seems to be insufficient in my opinion {particular readers would like know ‘how ‘b’ would serve your purpose? And ‘b’ is estimated after data collection}. In next line (by using same excellent reference) you say “To estimate MEC_90_, a minimum of 45 positive responses were required”, I wonder if that is enough? [because sample size is an important issue and though technical, little more is expected, I guess].

Male vs Female comparison in Table-1 {Characteristics of patients} was not irrelevant in my opinion [‘P’-value column]. To provide a description of baseline characteristics is entirely reasonable (since it is clearly important in assessing to whom the results of the trial can be applied), however, such statistical comparison [last ‘p-value’ column in Table 1] is not desirable at all. Purpose of Table-3 [Caudal epidural block characteristics and block complications of successful caudal block] is not understood. I think here also statistical comparison [last ‘p-value’ column in Table] may not be correct/desirable.

Other than these minor revision points, the article is acceptable, in my opinion/assessment.
